# Elimination of *Plasmodium vivax* Malaria in Azerbaijan

**DOI:** 10.4269/ajtmh.16-0173

**Published:** 2016-12-28

**Authors:** Suleyman Mammadov, Elkhan Gasimov, Rossitza Kurdova-Mintcheva, Chansuda Wongsrichanalai

**Affiliations:** 1 Republican Center for Hygiene and Epidemiology, Ministry of Health, Baku, Azerbaijan.; 2 Malaria and Other Vector-Borne and Parasitic Diseases, World Health Organization Regional Office for Europe, Copenhagen, Denmark.; 3 National Centre of Infectious and Parasitic Diseases, Sofia, Bulgaria.; 4 Independent Scholar, Bangkok, Thailand.

## Abstract

Azerbaijan in the south caucasus region of far southeastern Europe has a long history of malaria endemicity but just successfully eliminated local transmission. After a period of relatively stable malaria situation (1960–1970), the country witnessed an epidemic followed by a series of outbreaks of various magnitudes in the following two decades, all caused by *Plasmodium vivax*. Compared with 1993, the number of malaria cases in the country jumped 29 times in 1994, 123 times in 1995, and 571 times in 1996 at the peak of the epidemic, when 13,135 cases were officially registered. Incidence rate increased dramatically from 0.2/100,000 population in 1991 to over 17/100,000 population in 1996. Scaled-up malaria control led to the containment of the epidemic and to a dramatic decrease of malaria burden nationwide. Azerbaijan has applied contemporary, complex control and surveillance strategies and approaches and is currently in the prevention of reintroduction phase. This article describes Azerbaijan's public health experience in conducting malaria control and elimination interventions over several decades until 2013 when the country reached an important milestone—no indigenous malaria cases were recorded.

## Background

Azerbaijan is an eastern European country. All nations in this region have successfully eliminated malaria transmission. Azerbaijan is currently in the prevention of reintroduction phase but has gone through decades of high endemicity after seemingly successful control during the Global Malaria Eradication Campaign of the 1960s. However, due to the high receptivity of the territory, as well as to changes in vulnerability (seasonal migration of agricultural workers and mass displacement of refugees) and cessation of preventive activities, several outbreaks followed and in the mid-1990s the country had to fight an epidemic of the new era, *Plasmodium vivax* alone. This article describes historically malaria situation in Azerbaijan, country's malaria experience and evaluates the strategies and polices applied to control and eliminate *P. vivax*. The decades' long effort eventually led to a dramatic decrease of malaria burden, and zero indigenous cases recorded in 2013.

This article is based on several data sources, namely 1) national-level data from the Ministry of Health of Republic of Azerbaijan including materials on laws, regulations, orders, guidelines, reports; documents from the specialized services of malaria including reports, registers of malaria cases and foci, and maps and guidelines; documents from the Republican Center of Hygiene and Epidemiology, and of State Statistics Committee of Azerbaijan; 2) other related data from outside of the Ministry of Health such as demography, meteorology, economics, migration, and so on up to 2013; 3) scientific publications on malaria in Azerbaijan identified through PubMed and Google search and by screening through scientific publications from other sources; and 4) World Health Organization (WHO) reports of technical missions, records, as well as reports of the WHO Regional Office for Europe (WHO/EURO) meetings.

## Geography, Demography, and Health Systems

The Republic of Azerbaijan is located in south caucasus, the region on the edge of eastern Europe bordering southwest Asia. The country is bordered by the Greater Caucasus ranges to the north and the Caspian Sea to the east. Its neighboring countries are Russia, Georgia, Armenia, Iran, and Turkey. Azerbaijan has diverse landscapes, being largely mountainous but the eastern Caspian shoreline is characterized by lowland areas. As such, there is a wide range of climates throughout the country, from semi-desert and dry, subtropic, temperate to frigid. The temperature is 15°C on average in the plains but commonly drops below zero in high mountains. In July, the average temperature reaches 25–27°C in Aran regions and 5°C in the mountain regions. Precipitation also varies across the territory, from 200 to 1,700 mm per year.

According to Azerbaijan's 2013 demographic data, the country has a population of 9.4 million. Slightly over half of them live in urban areas.^1^ The health system in Azerbaijan is highly centralized and hierarchical. The Ministry of Health has the ultimate responsibility for the overall health of citizens, but the Ministry has limited means to influence health-care providers at the local level. The Ministry runs central-level institutions and tertiary level (Republican) hospitals, research institutes and the sanitary epidemiological services. However, local governments own district hospitals, polyclinics, and specialized clinics. These local health facilities are run by district health authorities, who follow the national health policy, but are financially dependent on the local governments. The Ministry is represented at the local level by the district health authorities. Malaria control and elimination activities are coordinated by the sanitary epidemiological services at central and district level.

## Malaria Situation Before the WHO Global Malaria Eradication Campaign of the 1960s

*Plasmodium vivax*, *Plasmodium falciparum*, and *Plasmodium malariae* were long found in the country. *Plasmodium falciparum* malaria, with a case fatality rate of 50–70%, was recorded in some settlements such as Guba-Khachmaz (districts of northeast mountainside of the Greater Caucasus) and south Mugan, during 1920s.^2^,^3^ Large-scale malaria epidemics occurred in 1917–1921 and more than 500,000 cases were registered during 1934–1935.

There was a progressive decline in malaria incidence—from 125.9/100,000 population in 1950 to 9.0/100,000 population in 1956 and to 0.27/100,000 population in 1960. By 1959, malaria morbidity was registered only in 29 districts, with 2,017 cases (89.9% of all cases throughout the country) registered only in Masalli, Lenkoran, and Imishli districts.

No local transmission has been documented since 1957 for *P. malariae* and since 1960 for *P. falciparum*.^4^ These species were successfully eliminated. However, *P. vivax* had never been eliminated and later staged comebacks in Azerbaijan after the 1970s.

## Persistent *P. vivax*

### Locally acquired cases.

**Figure 1.**
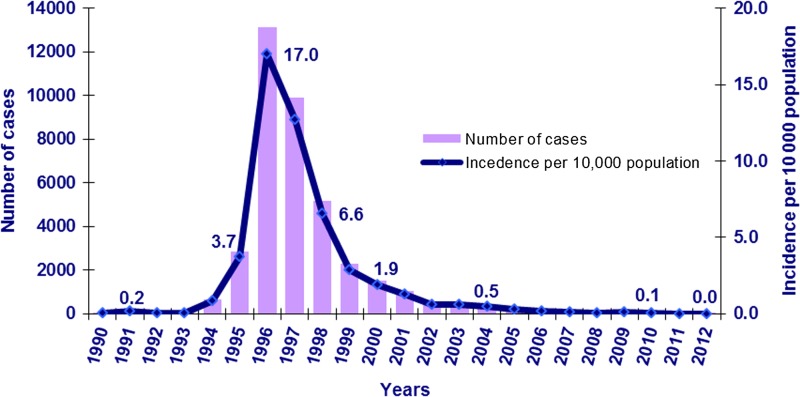
Malaria cases and malaria incidence per 10,000 population, 1990–2012.

Outbreaks of limited scale were registered during the 1970s and 1980s, but from 1994, a serious epidemic occurred. Compared with 1993, the number of malaria cases in the country jumped 29 times in 1994, 123 times in 1995, and 571 times in 1996. In 1996, a total of 13,135 cases were officially registered (Figure 1

). Malaria incidence had increased dramatically from 0.2/100,000 population in 1991 to over 17/100,000 population in 1996 (Figure 1). Presumably, the real numbers were much higher because malaria cases in some organized population groups and some occupied territories were not included in the national data.

Factors contributing to the epidemic included worsening of socioeconomic conditions, cessation of preventive activities, changes in agricultural practices affecting mosquito habitats, and increase in the seasonal migration of agricultural workers. The most important precipitating factor was likely the mass displacement of nearly 1 million refugees and internally displaced people (IDP) as a result of armed conflicts. The majority of these refugees and IDP were previously malaria naive (most of them had lived in mountainous and foothill regions where local malaria cases had been absent for over 40 years), settled in temporary camps in regions with existing residual malaria foci. Poor standard of living and inadequate health services led to explosive outbreaks in the camps, which spread countrywide because of uncontrolled migration within the country.

As a result of accelerated large-scale malaria control interventions with mobilization of both internal and external resources, the number of malaria cases steadily declined from 1997, and fell to zero by 2013.

### Case distribution.

The malaria transmission season in Azerbaijan runs from May to October. The pattern of the seasonal distribution of malaria cases did not change in the past several decades and was not affected by the decline in malaria transmission. Peak malaria transmission occurs during June–August.

Indigenous malaria cases that occurred in the first 5 months of the calendar year can be largely considered as cases of *P. vivax* with long incubation, which have contracted malaria in the previous malaria season. The proportion of such cases among the total number of cases in the country is around 30% per year. A great attention is paid to those cases as to potential sources of spreading.

Malaria is detected in all age groups, but the most affected are those 15 years old and above. For the past 15 years, there has been a progressive decline of malaria being preponderance in men (60%) eventually reaching parity with women. This probably suggests the decrease in the risk of infection outside of their places of residence, as men are the most mobile group of the population.

### Imported malaria.

Importation of malaria cases to Azerbaijan has been minimal. Proportion of imported cases became prominent recently as the locally transmitted cases dropped to only a small number. In the past decade, there were less than 20 imported cases with *P. falciparum* slightly outnumbered *P. vivax*. In 2013, all cases reported in the country were because of imported *P. falciparum* malaria.

### Glucose-6-phosphate dehydrogenase deficiency.

Based on a study published by Azerbaijan Research Institute of Hematology and Blood Transfusion in 2007, the highest prevalence of glucose-6-phosphate dehydrogenase (G6PD) deficiency detected was 25%.^5^ The issue of G6PD deficiency came to the attention following the outbreaks in 1970s.^6^

At the time, data on G6PD deficiency were available only from two districts—Masalli and Barda, both in lowland/plain areas. No more than 7% G6PD deficiency was recorded at those sites. Expanded surveys were then conducted in 18 districts covering over 24,000 people during 1971–1973 and found that G6PD deficiency varied from 1.9% to 7.0% in submountainous and mountainous districts to 6.0–38.4% in lowland/plain districts, which were much higher than previously thought.^6^ High rates of G6PD deficiency where malaria most often occurred thus challenged the necessary administration of primaquine to patients and populations.

### Anopheline vectors.

**Figure 2.**
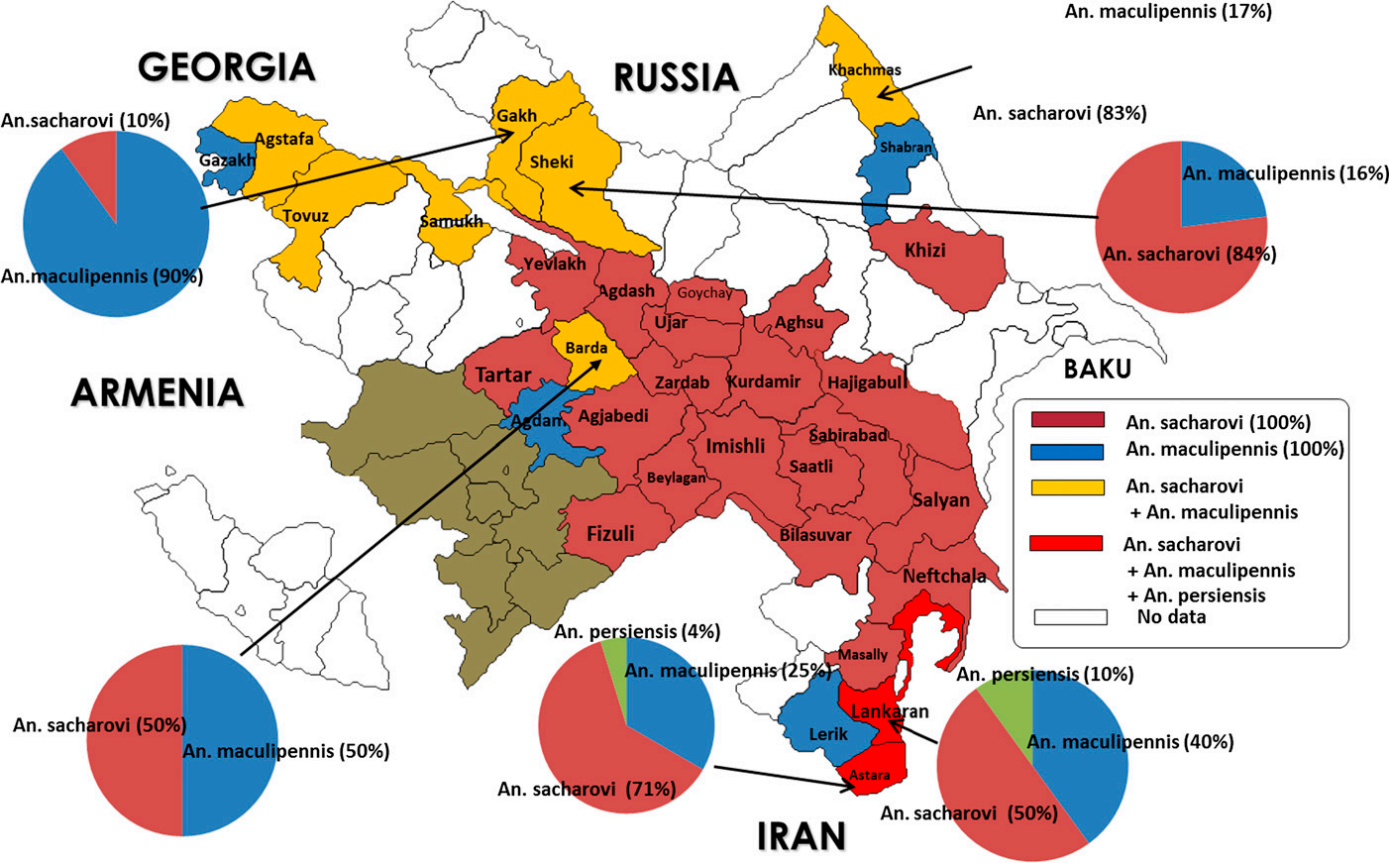
Distribution of *Anopheles maculipennis* complex by district in Azerbaijan (by A. B. Zvantsov, unpublished data).

Primary malaria vectors in Azerbaijan are *Anopheles maculipennis* and *Anopheles sacharovi*. *Anopheles sacharovi* is the main vector of the Kura-Araz valley, whereas *An. maculipennis* is that of the Greater and Lesser Caucasus. Both of them bite at sunset and early morning hours and rest in homes or cattle sheds. *Anopheles maculipennis* tends to breed in warm, clean water reservoirs with algae but *An. sacharovi* prefers salty, warm water. Both are highly susceptible to alpha cypermethrin, cyfluthrin, and deltamethrin. The absence of resistance to pyrethroids among major malaria vectors in the country is one reason for the ability to halt malaria transmission in all active foci without a complicated process. *Anopheles hyrcanus*, *Anopheles plumbeus*, and *Anopheles claviger* were also believed to contribute to malaria transmission in the country in the past; however, a number of research studies conducted during 2009–2013 did not suggest such roles for any of these three species. Findings of these studies are summarized in Figure 2

.

## Postepidemic Malaria Control and Elimination

### Policy, commitment, and finance.

Following the start of the 1994 *P. vivax* epidemic, the Ministry of Health became aware of possible deterioration of the situation and so considerable efforts were made to scale up malaria control. However, at that time only limited funds were available. Until 1998, the Ministry focused solely on Mass Drug Administration (MDA) and health education. By 1997, about one-sixth of the population was subjected to seasonal weekly chloroquine prophylaxis and the first signal of a decrease in malaria endemicity was observed with a 25% reduction of cases (9,911 cases in 1997 versus the 13,135 cases in 1996). The prospect of more complete reductions, however, was not realistic until a well-coordinated national plan with intersectoral collaboration was established with external assistance in 1998 and additional funding provided.

In July 1998, the Roll Back Malaria Partnership was established with participation by WHO, United Nations International Children's Emergency Fund, and International Federation of Red Cross and Red Crescent Societies, Medecins Sans Frontieres Belgium, and Eni, an Italian oil and natural gas company, to provide support to the specialized malaria control services of Azerbaijan Ministry of Health. In 1999, within the framework of this partnership, Eni agreed to contribute US$760,000 to finance, though WHO/EURO, a 3-year malaria control plan drafted by the Ministry of Health.

The objective of the plan was to reduce malaria incidence to a level of only sporadic cases by the year 2004 and to avoid social and economic consequences of the malaria burden. The plan aimed to scale-up malaria control by applying a comprehensive approach to accelerate vector control and surveillance. The main strategic directions of the program were to improve the capacity for and access to early diagnosis and adequate treatment of malaria within the primary health-care system, promote cost-effective and sustainable vector control, strengthen the overall malaria control capacity including research capability, and reinforce malaria surveillance as well as antiepidemic response. In addition, the plan aimed to increase community awareness and engagement and to enhance intersectoral collaboration.

According to the Ministry of Health, it was estimated that implementation of the program had protected from 1999 to 2001 around 1.5 million people living in 40 districts of Azerbaijan against malaria. In view of remarkable results achieved in malaria control during the last several years, the Government endorsed in 2005 the Tashkent Regional Declaration “The Move from Malaria Control to Elimination” in the countries of the WHO European Region^7^ and embarked upon the implementation of the National Malaria Elimination Strategy for the years 2008–2013.

The ultimate goal of the new national strategy was to interrupt malaria transmission by 2013 and to be followed by certification of malaria elimination. In areas where malaria had been eliminated, attention was to be given to maintaining the malaria-free status. Particular emphasis was also placed on tackling the growing problems associated with imported malaria.^7^

### Vector control: strategies and coverage.

#### Indoor residual spraying.

Indoor residual spraying (IRS) was the main vector-control measure applied in the country with the use of synthetic pyrethroids. Since 2009, alpha cypermethrin 0.05% has been used. IRS is applied to communities free of charge twice a year: the first round in April to early May and the second round in June-August. Procurement of insecticides and IRS was scheduled in advance, at the end of preceding year based on an assessment of the malaria situation. Insecticide stockpiles for urgent deployment in case of outbreaks are kept at the Republican Center of Hygiene and Epidemiology.

Only small-scale IRS campaigns using mainly organophosphates had been implemented before 1998. Through the external support of the roll back malaria initiative, large-scale campaigns using biodegradable insecticides of the synthetic pyrethroid type (cyfluthrin and alpha cypermethrin) were made during 1999–2000. In 2010, Azerbaijan suffered from heavy floods covering huge area especially malaria-endemic zones. To prevent resurgence of malaria, the Ministry of Health stepped up IRS to cover 1,250,000 people (5- to 10-fold increase) during that year.

#### Larval control.

*Gambusia affinis*, introduced in 1930,^3^ was widely used for mosquito control all over the country. The fish adapt well to different types of water reservoirs in almost all landscapes of Azerbaijan. No other chemical or biological means have been used for larvae control. At some point in mid-1990s, mosquito breeding sites were treated with oil, but this was only for a brief period due to concerns of interference with native predators of the habitats and biodiversity.

#### Bednet distribution.

Long-lasting insecticide-treated nets (LLINs) distribution is directly connected with projects under the Global Fund to Fight AIDS, Tuberculosis and Malaria (GFATM), which began support to Azerbaijan malaria control in 2009. Because of limited supplies, the primary target population for LLIN consists of pregnant women and children under 5 years old residing mainly in refugee camps. LLINs were well accepted by the communities. During 2009–2012, 50,000 LLINs were distributed.

### Case management.

#### Laboratory diagnosis.

Malaria diagnosis is based on microscopy and is free of charge in the public sector. There is a wide network of diagnostic laboratories in the public sector throughout the country. Rapid diagnostic test has not been considered at any time.

Altogether 233 laboratories all over the country have the capability to diagnose malaria. They have an established program for capacity strengthening of microscopists. They participate in the National Scheme for External Quality Assurance (EQA) of malaria microscopy. All blood smear positive samples detected in any of the laboratories and 10% of all negative samples are sent to the reference laboratory for confirmation and rereading during the monthly quality assurance (QA) cycles. The Parasitology Laboratory of the Republican Center of Hygiene and Epidemiology serves as the national reference laboratory of malaria. The Laboratory has successfully participated in an international EQA Program (August/September 2014) and received a certificate of competency.

#### Treatment of malaria.

Antimalarial drugs are procured at the central level by the Ministry of Health and dispensed by treatment facilities in the public sector only. Private sector pharmacies are strictly prohibited from carrying any antimalarial drugs.

All parasitologically confirmed cases of malaria receive a full treatment course for free according to the national treatment guidelines (chloroquine 25 mg base/kg body weight [bw] divided into 3 days: day 1: 10 mg base/kg bw, day 2: 10 mg base/kg bw, day 3: 5 mg base/kg bw and administered per os, and primaquine once a day at 0.25 mg base/kg bw per os is administered with food for 14 days).^7^

The national protocol recommends against primaquine in pregnant women, during breast-feeding or in children under 4 years old. In pregnancy, a full treatment course with chloroquine is required. Administration of primaquine should only be started 3 months after delivery.

In case of known G6PD deficiency, primaquine 0.75 mg base/kg bw should be given per os once a week for 8 weeks. The directly observed therapy (DOT) protocol is applied. If any signs of hemolysis are observed, the drug must be discontinued.

In case of chloroquine-resistant *P. vivax,* the alternative to chloroquine is amodiaquine given in combination with primaquine. For uncomplicated *P. falciparum* malaria, the first-line therapy is artesunate plus sulfadoxine–pyrimethamine. Treatment of *P. malariae* is with chloroquine over 3 days.^7^

### Mass Drug Administration.

MDA implies the administration of antimalarial drugs to every individual in a defined population. In Azerbaijan, MDA is considered, along with IRS and other control measures, in the following circumstances in areas with limited, seasonal malaria: 1) when small foci of malaria continue to exist after transmission has been interrupted elsewhere, 2) when an outbreak has been reported, and 3) when IRS fails to fully interrupt transmission.^7^

#### Primaquine.

For the first time, MDA with primaquine was used in Azerbaijan in 1971–1975 in response to the outbreak of *P. vivax* that occurred after decades of a presence only of few residual foci. The main features of the outbreak were 1) mono-infection with *P. vivax*, 2) mixed parasite populations of both short and long incubation, and 3) strong resistance of the main vector, *An. sacharovi*, to dichlorodiphenyltrichloroethane. In this situation, it was decided to implement mass treatment of the affected population with primaquine antirelapse therapy. Besides a high coverage of primaquine administration, prevention of adverse effects of the drug in people with G6PD deficiency was deemed necessary. Over 230,000 people were covered.^6^ Generally, mass primaquine prophylactic treatment (MPPT) in Azerbaijan involved the whole population of a settlement, with the exception of children < 4 years old, pregnant women, and breast-feeding mothers. G6PD test was not obligatory and not conducted. Participants take primaquine DOT by medical staff. Participants were instructed to stop primaquine if any sign of adverse effects (low back pain, changes in the urine color, icteric sclera, etc.) developed. No death or severe adverse events related to primaquine use has been recorded despite the wide use of the drug for both treatment and chemoprevention.

To avoid possible adverse effects in areas with high prevalence of G6PD deficiency, primaquine was administered on the so called “partial load” mode. According to the “partial load” mode guidelines, primaquine was given on days 1–4, interrupted on days 5–7, then continued on days 8–17.^6^

For 15 years, MPPT was implemented several times in a number of districts with an average coverage of 92%.The campaigns have been successful showing primaquine to be a highly effective tool for controlling *P. vivax* outbreaks/epidemics leading to several-fold reduction in morbidity in a single malaria season. The Azerbaijan experience supports the dependence of program success on high coverage (over 90% of targeted population), administration of primaquine under DOT, and careful monitoring of primaquine adverse effects. It is well accepted that MDA with primaquine alone without implementation of companion malaria control measures, in particular vector control, cannot achieve interruption of transmission.^6^

#### Chloroquine.

In 1996–2000, chemoprevention with chloroquine during the transmission season was also implemented (Table 1 ). However, since 2000, this preventive approach has been discontinued.

### Therapeutic efficacy.

In 2001, Azerbaijan began monitoring therapeutic efficacy of chloroquine based on WHO protocol.^8^ Among the 153 patients included in the study, 17, 28, 35, 10, 4, 20, and 39 were recruited from the districts of Aghjabadi, Barda, Baku, Beylagan, Imishly, Sabirabad, and Saatli, respectively. The mean age (range) of the patient was 30.8 (6–/85) years. All patients were febrile on day 0 and/or reported history of fever within 24 hours. Parasitemia geometric mean (range) was 3,333 (200–80,080)/μL. The average (range) of the chloroquine dose was 24.4 (20.1–27.9) mg/kg. The duration of the chloroquine treatment was 3 days. One hundred and forty-three of patients were evaluable on day 28. There were 100% successful clinical outcomes. The results indicated that chloroquine was fully effective over the first 14 days, and the combination of chloroquine and primaquine was fully effective over 28 days.^8^ No therapeutic efficacy studies have been conducted since then.

### Epidemiological surveillance.

#### Surveillance system.

The Ministry of Health is responsible for the planning, implementing, and monitoring of malaria control/elimination activities. Five malaria case reporting forms are used to capture key information, which is the basis for building a national malaria database. These forms are 1) Urgent Notification, 2) Epidemiological Investigation (patient data, possible source of infection, related malaria focus data, and result of case investigation), 3) Laboratory Report (microscopy findings), 4) Malaria Focus Monthly Report (population and malaria cases, demographic distribution, active/passive case detection (PCD) results including time between onset of illness and detection), and 5) Vector Control Report.

Since 2009, malaria cases are reported in electronic integrated diseases surveillance system (EIDSS). EIDSS is an electronic diseases surveillance system that integrates both human and veterinary case data collection and covers demographic information, geographical information, laboratory analyses, sample tracking, epidemiological analyses, clinical information, and response measures. This cohesive, secured database is continuously synchronized among all EIDSS sites within the country. The database is accessible by appropriate organizations; data can be retrieved almost real time and are also available for historical analysis.

#### Case detection.

Azerbaijan adopted the WHO-standardized case definitions for malaria in 2008. For elimination purposes, a malaria case is a person in whom, regardless of presence or absence of clinical symptoms, malaria parasites have been confirmed by quality-controlled laboratory diagnosis.

In Azerbaijan, both PCD and active case detection (ACD) are used.

**Figure 3.**
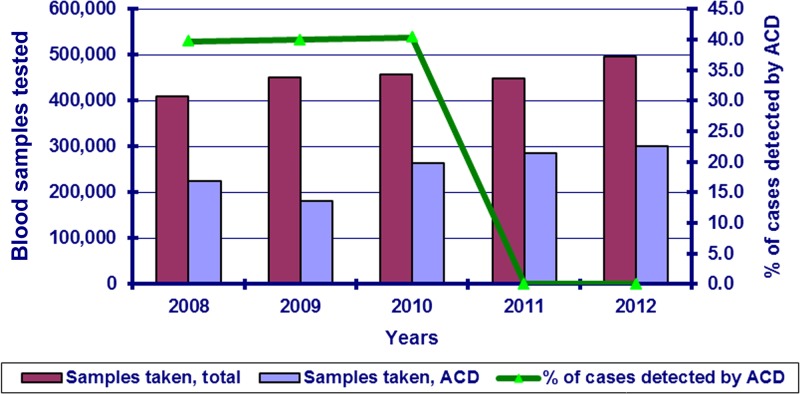
Blood samples tested and malaria cases detected by active case detection (ACD), 2008–2012.

PCD: Malaria blood slides are taken for parasitological examination from all febrile patients and clinically suspected malaria cases. According to the existing regulation, patients with fever who visit medical facilities all over the country are subjected to malaria screening.
ACD: Approximately 30–40% of all blood samples tested in the country are collected by ACD (Figure 3). ACD is carried out by the primary health-care services (outpatient clinics, health posts) based on recommendations of the Republican Center of Hygiene and Epidemiology. ACD activities are under the control of respective district Center of Hygiene and Epidemiology.

##### Proactive ACD.

Regular ACD is carried out during transmission season in risk areas by household visits once every 2 weeks. All active foci (including new, endemic, and residual) and any areas with the last local case registered within last 5 years are considered risk areas. During household visits, blood is taken from all individuals with current or recent febrile illness to test for malaria (and treat if positive).

##### Reactive ACD.

During an epidemiological investigation, if a new malaria case is detected, all the family members of the patient and of all residents of the focus are examined. Ideally, according to the existing instruction blood samples have to be taken from 100% of focus residents. However, in some cases, it is not possible to achieve the target (out of home, few cases of refusal, etc.) but at least 80% of the focus population should be screened.

To cope with the issue of imported malaria, Azerbaijan developed guidelines for surveillance of imported malaria cases with the assistance of WHO; the document is under revision (2014).

#### Malaria foci and their management.

Interventions during elimination programs are based on the concept of a malaria focus, assuming that transmission is focal and no longer homogeneous across the country. Azerbaijan adopted the WHO definition of malaria focus (2008): “A focus is a locality such as a town, a village or other defined geographical area in which there are Anopheles breeding sites, feeding and resting places, and people exposed to biting by the vectors. It is described as a defined and circumscribed locality situated in a currently or formerly malarious area and containing the continuous or intermittent epidemiological factors necessary for malaria transmission.” Foci are classified depending on 1) their age—residual versus new and 2) the presence of malaria transmission—nonactive versus active versus potential (also based on WHO classification). Regular monitoring of the foci is carried out by district sanitary epidemiological authorities and the recorded status is available locally and nationally.

MDA was also applied. In 2011, MDA with primaquine was implemented in seven districts with residual foci, covering over 10,000 people. The operation was successful and no serious adverse events were recorded despite inclusion of villages with the highest prevalence of G6PD deficiency (Aghsu and Goychay districts).

#### Entomological surveillance.

Entomological monitoring is conducted by district sanitary epidemiological teams on selected control points and includes mosquito species composition, density, phenology and distribution, registration and mapping of breeding sites. These data supplemented by the information of the meteorological monitoring and analyses of weather conditions and climatic trends allows determination of the periods of the potential malaria season.

### Confirmation of interruption of local transmission.

The conclusion that the interruption of the chain of local malaria transmission by *Anopheles* mosquitoes throughout the country was made on the basis of data of zero indigenous cases registered in 2013 and gradual decrease and clearing up the malaria foci. In 2012, two out of three reported cases were indigenous cases. In 2013 and 2014, only imported cases were reported. In Azerbaijan, adequate malaria surveillance and response system is fully functional across the entire territory of the country. Azerbaijan's incessant effort to search for the last case of locally transmitted malaria is also a key to the elimination success. Annual blood examination rate (ABER; or the number of blood smears examined in a year × 100/total population) peaked at 7.3% in 1998 and continued at 5.5–6.5% during 2002–2007, when the total number of malaria cases fell below 1,000. From 2008, when the total number of cases dropped to under 100, ABER remained at > 4% coverage even during 2013 and 2014 when no indigenous case was detected. These data support the conclusion of having zero indigenous cases nowadays. Early case detection, treatment, and epidemiological investigation bring about timely elimination of the sources of infection. The surveillance information system (case and foci registers, epidemiological investigation forms, reports, and etc.) and monitoring and evaluation provide reliable information indicating the interruption of the local transmission. Several indicators had been used for the assessment of the efficacy of antimalarial measures and evaluation of progress made toward interruption of local transmission such as: timelines between diagnosis and reporting, treatment and epidemiological investigation, number of active foci reported/year, proportion of cases reported to surveillance system, total population at risk within country, proportion of cases confirmed by microscopy, proportion of cases treated according to guidelines, proportion of at-risk households/proportion of reported active foci that were sprayed, proportion of known/potential breeding sites treated with chemicals/fish, proportion of breeding sites positive for larvae, etc.

### Cross-border cooperation.

Now that Azerbaijan has progressed to elimination phase, cross-border collaboration is very important to avoid reintroduction by spreading of malaria between countries and neighboring regions.^7^

**Figure 4.**
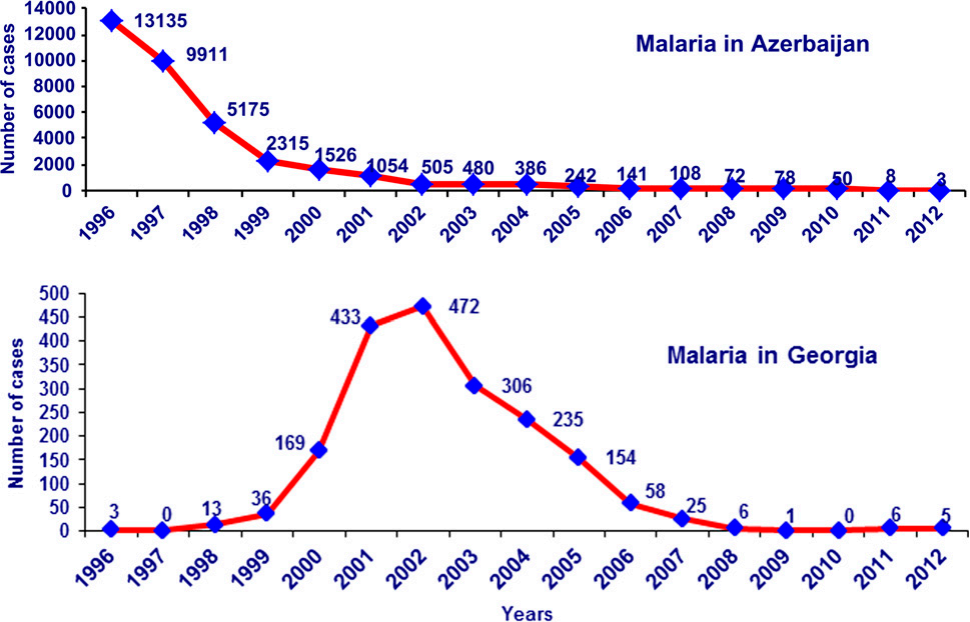
Dynamic of malaria cases in Azerbaijan and Georgia, 1996–2012.

Azerbaijan and Georgia committed themselves to eliminate malaria by endorsing the Tashkent Declaration in December 2005. In 2008, both countries developed National Malaria Elimination Strategies and shifted national malaria programs from control to elimination. In 2012, only three cases were registered in Azerbaijan, out of which only two were indigenous. Malaria transmission in Georgia was interrupted in 2010 and in 2011 and 2012 two introduced *P. vivax* cases were reported as a consequence of malaria importation from India in an area bordering Azerbaijan (Figure 4

).

Seven districts of Azerbaijan are bordering Georgia. A total population over 600,000 people representing 20 ethnic groups, including Azerbaijanis, Georgians, Russians, Avars, and so on, live there. On the Georgian side, over 416,000 people, mainly of Georgian and Azerbaijani ethnicities reside in six districts bordering Azerbaijan. The main malaria vectors are the same (*An. maculipennis* and *An. sacharovi*) and transmission season is from July to October in both countries.

**Figure 5.**
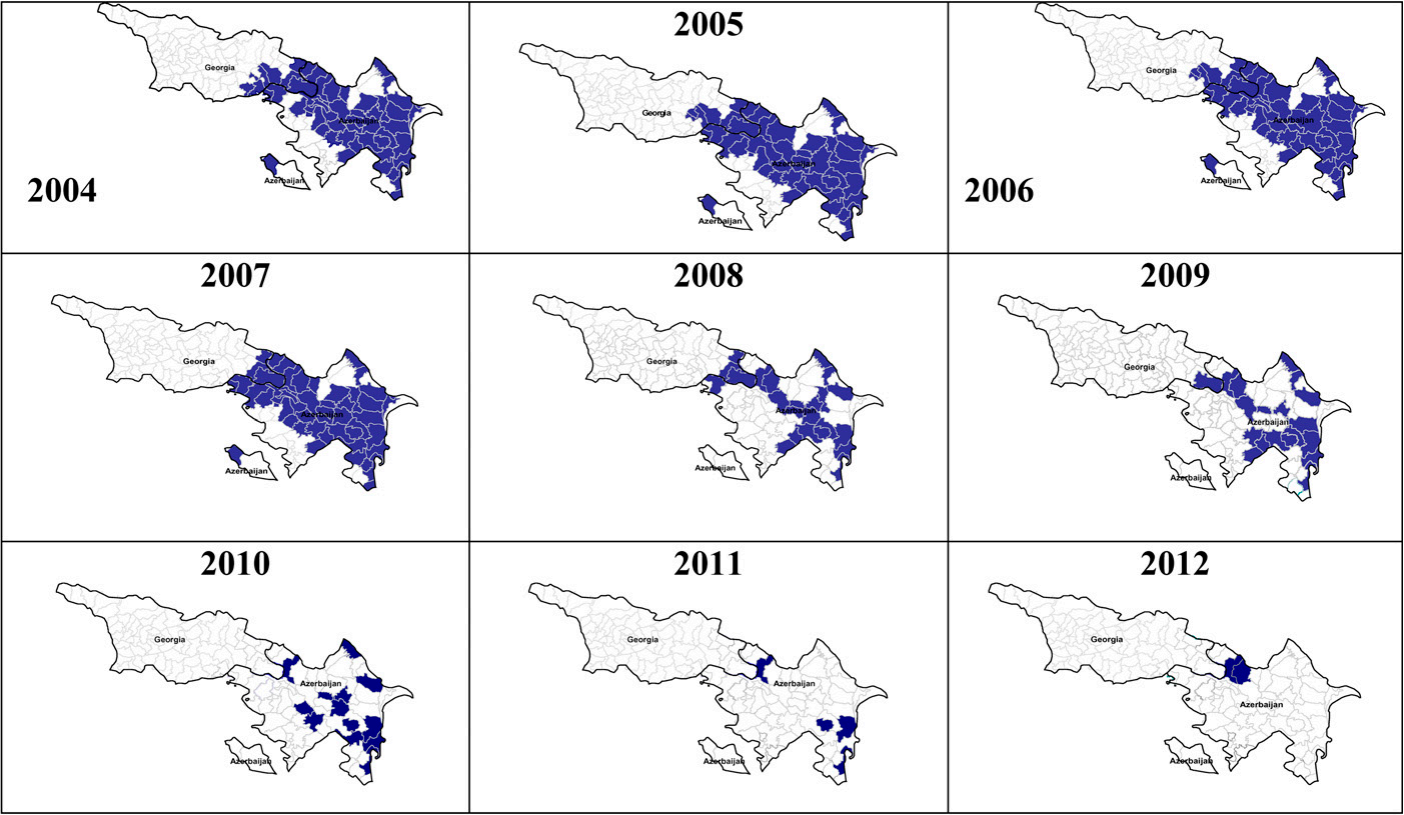
Distribution of malaria by districts of Azerbaijan and Georgia, 2004–2012.

Close political, economic, and cultural ties between countries, freedom of cross-border movement, mixed ethnic composition of residents, as well as malaria epidemiological trends suggest the inter-dependence of malaria situation of these two countries (Figure 5

).

Therefore, it was deemed necessary to establish structured and well-organized cross-border collaboration between two countries. The first inter-country coordination meeting on malaria elimination between Azerbaijan and Georgia was held in Baku in March 2009. The Joint Statement on inter-country cooperation on malaria elimination between Azerbaijan and Georgia was endorsed by the Ministries of Health, the GFATM-funded malaria projects of both countries, and WHO/EURO. Joint cross-border activities were launched in May 2010 with the participation of all stakeholders. The joint operation progresses well.

### Monitoring of progress toward malaria elimination.

To ensure good progress toward elimination, the program applied a set of indicators covering five key areas:

1. Enabling environment: This is to ensure that the legal/regulatory frameworks remain in place and regulatory authorities remain in function thus malaria continues to be a notifiable disease.
2. Vector control: This includes coverage of at-risk households and reported active foci that had been sprayed, management of known/potential breeding sites, and routine assessment of breeding sites positive for larvae.
3. Epidemiology: The program keeps reviewing annually the number of active foci reported, number of cases within foci, and proportion of foci correctly classified.
4. Case management: The program ensures confirmation of diagnosis by microscopy, correct treatment and presence of microscopy QA/QC.
5. Surveillance: Time between diagnosis and notification/reporting reporting of cases to the surveillance system as well as the number of reported cases and the remaining population at risk are monitored.

## Conclusions

In the 1990s, there was a massive return of *P. vivax* malaria to many countries in eastern European, central Asia, and the caucasus region. The affected countries joined forces to bring down the level of transmission with support from the Roll Back Malaria initiative under WHO guidance. The scaled-up intervention measures through vector control, case management, and surveillance led to rapid and marked reduction of the malaria burden. Azerbaijan was among those countries that applied a complex approach for containing the *P. vivax* epidemics and protecting the health of the population. Presently the national goal, which is interruption of local malaria transmission by 2013, has been achieved. This would not have been possible without the following contributing factors:

1. Strong political and financial commitments toward malaria elimination were key to success. Support rendered by international and bilateral organizations, particularly WHO and GFATM proved beneficial. Further reenforcement of sustainable funding through high-level commitments and other support helped to fulfill the elimination goal. This was a result of visible impact of the comprehensive antimalaria activities conducted by the National Malaria Elimination Program in cooperation with the General Health Services.
2. Azerbaijan is also fortunate to be adequate with existing infrastructure, skilled and motivated personnel, excellent community cooperation, and program flexibility that allows modification/revision of existing strategies to ensure higher efficacy of available technologies and tools. The country has also had experience and demonstrated feasibility to practically “eliminate” malaria (or suppress it to a low level) in the country.
3. As described below, several other contributing factors, mostly of socioeconomic nature likely helped to further diminish transmission.^1^

There has been a sharp decrease in land areas dedicated for rice cultivation that used to be responsible for numerous mosquito breeding sites. Rice production dropped from 8,300 ton in 2005 to 3,900 ton in 2012. There was a drastic fall of agricultural land use for cotton production in almost 10-folds. This led to a reduction in the number of mosquito breeding sites caused by irrigation for cotton plantation and also obliterated large-scale seasonal migration of the agricultural labor force, usually from nonendemic to malaria-endemic areas, for cotton harvesting. Similarly for the silk industry, following considerable downsizing in recent years, only four districts on the territory of malaria-endemic zone remain engaged in silk worm rearing of a limited scale. Absence of silk worm rearing practices favors the use of insecticides inside the premises for malaria prevention and control. At the same time, there was an appreciable increase in cattle population in the private sector of about 60% from 1996 to 2012, thus facilitating the deviation of anopheline mosquito from feeding on human hosts to animals. In addition, malaria situation on the territory of neighboring states has been improved following implementation of similar strategy of malaria elimination (Georgia, Iran, and Turkey) or having already achieved malaria-free status (Armenia). Lately there has also been a new trend for the preferred destinations of Azerbaijanis traveling abroad being malaria-free areas like the United States, United Kingdom, European Union countries, and the Russian Federation thus minimizing the chance for malaria reintroduction.

On the other hand, there are a few factors, which still contribute toward malaria receptivity and vulnerability. Economic growth and improvement in the transportation system increased in the turnover of passengers (air, rail, and road) by 3.2-folds during 1995–2012.^1^ Such a situation may facilitate very rapid proliferation of infection throughout the country within a very short period of time. Besides, irrigation system in the country is still inadequate resulting in the loss of water during its transit thus potentially creating breeding sites for *Anopheles* mosquitoes.

Now that local *P. vivax* transmission has been interrupted, Azerbaijan is directing more effort toward prevention of resurgence. Malaria receptivity is much confined to the rural areas; transmission potential is very limited in the urban areas. People who are engaged in agricultural activities, particularly those depending on irrigation system, remain at some risk. Under low the transmission level characteristic of Azerbaijan, practically all age groups and both genders are vulnerable to malaria infection, particularly infants and pregnant women.

Military personnel, border guards deployed in areas of military conflict, and refugees, particularly those still living under precarious conditions, are especially at increased risks. Azerbaijan national malaria control program also emphasizes the need for a robust level of vigilance for imported malaria.

On the whole, the experience of Azerbaijan shows that malaria resurgence is possible due to reasons related to receptivity and vulnerability. It has demonstrated that a strong public health system infrastructure, political support, and rapid resource mobilization are needed for the successful containment of epidemics. Dealing with *P. vivax* is known to involve a lengthy process. Sustained funding, even over a decade later, has proved to be useful for preventing any return of the outbreaks, thus making further progress toward elimination. Azerbaijan national malaria control program is making every effort toward those goals and is expecting the whole Republic to be officially certified for malaria elimination soon.

## Figures and Tables

**Table 1:** Use of chloroquine for preventive treatment

Years	No. of settlements involved	No. of people covered by seasonal chemoprophylaxis
1996	455	494,784
1997	760	1,020,087
1998	437	408,106
1999	336	318,416
2000	3 (in refugee camps)	20,000
